# Association of perceived life satisfaction with attitudes toward life-sustaining treatment among the elderly in South Korea: a cross-sectional study

**DOI:** 10.1186/s12904-022-01072-6

**Published:** 2022-10-17

**Authors:** Il Yun, Hyunkyu Kim, Eun-Cheol Park, Suk-Yong Jang

**Affiliations:** 1 Department of Public Health, Graduate School, Yonsei University, Seoul, Republic of Korea; 2 Institute of Health Services Research, Yonsei University, Seoul, Republic of Korea; 3 Department of Preventive Medicine, Yonsei University College of Medicine, Seoul, Republic of Korea; 4 Department of Psychiatry, Yonsei University College of Medicine, Seoul, Republic of Korea; 5 Department of Healthcare Management, Graduate School of Public Health, Yonsei University, 50-1 Yonsei-to, Seodaemun-gu, Seoul, 03722 Republic of Korea

**Keywords:** Life-sustaining treatment, Well-dying, End-of-life care, Life Satisfaction, Self-determinants right

## Abstract

**Background:**

Amidst rapid population aging, South Korea enacted the Well-dying Act, late among advanced countries, but public opinion on the act is not still clear. Against this background, this study aims to: 1) investigate factors affecting elderly individuals’ attitude toward life-sustaining treatment, and 2) examine whether attitude toward life-sustaining treatment is related to their perceived life satisfaction.

**Methods:**

Data from the 2020 Survey of Living Conditions and Welfare Needs of Korean Older Persons were used. There were 9,916 participants (3,971 males; 5,945 females). We used multivariable-adjusted Poisson regression models with robust variance to examine the association between perceived life satisfaction and attitude toward life-sustaining treatment and calculate prevalence ratios (PR) and 95% confidence intervals (CI).

**Results:**

After adjusting potential confounders, the probabilities that the elderly who were dissatisfied with their current life would favor life-sustaining treatment were 1.52 times (95% CI: 1.15–1.64) and 1.28 times (95% CI: 1.09–1.51) higher for men and women, respectively, than the elderly who were satisfied. In addition, attitudes in favor of life-sustaining treatment were observed prominently among the elderly with long schooling years or high household income, when they were dissatisfied with their life.

**Conclusions:**

Our results suggested that for the elderly, life satisfaction is an important factor influencing how they exercise their autonomy and rights regarding dying well and receiving life-sustaining treatment. It is necessary to introduce interventions that would enhance the life satisfaction of the elderly and terminally ill patients and enable them to make their own decisions according to the values of life.

**Supplementary Information:**

The online version contains supplementary material available at 10.1186/s12904-022-01072-6.

## Introduction

The increase in discussions on end-of-life care, including hospice palliative care and withdrawal of life-sustaining treatment (LST), is closely linked to population aging [1]. In particular, Korea is the fastest aging country in the world, and as of January 2022, the proportion of the elderly population is close to 17.5% [2], which falls within the classification of an aged society.

Rapid population aging has caused many problems, such as making elderly patients and their families suffer pain and disability for a long time until death, and greatly increasing the economic burden of LST [3]. Against this background, interest in process of choosing death or LST according to the right to self-determination and actively preparing for death throughout life has increased worldwide [4]. Similarly, in Korea, a new turning point in end-of-life care has arrived with the ‘Hospice/Palliative Care Act’ and the so-called ‘Well-dying Act’ which came into force in 2018 [1, 5, 6]. In Korean Society, well-dying is defined as a concept with the attributes such as reflection on death, acceptance of death, advance care planning, and transcendence [7]. The main content of this act is that LST against the patient's will can be stopped at any time [8].

LST is defined as any treatment that serves to prolong life without reversing the underlying medical condition and includes processes such as mechanical ventilation, renal dialysis, chemotherapy, antibiotics, and artificial nutrition and hydration [9, 10]. The relevant Acts aim to protect the best interests of the patients and respect their self-determinants rights [11]. In countries where well-dying related legislation was implemented earlier, there have been numerous studies and interventions on LST. Patients’ perceptions of end-of-life care [12, 13], as well as related physicians’ orders [14, 15], and ethical considerations [16, 17] were discussed. The issue of forgoing LST is par important for critically ill patients or elderly people on the brink of death. Thus, a number of studies have reported on LST preferences for patients in the intensive care unit or suffering from terminal illness [18–20].

However, in Korea, not long after the Well-dying Act was enacted, societal consensus is still in the process of developing, so there are not many preceding studies examining the perceptions of seriously ill patients and the elderly toward preparation for death or receiving LST [1, 11]. Therefore, this study aimed to investigate factors affecting decision making about LST among the Korean elderly and, in particular, examine the association between perceived life satisfaction and attitudes toward LST.

## Methods

### Data and study population

The data analysed in this study was taken from the 2020 Survey of Living Conditions and Welfare Needs of Korean Older Persons, a nationwide time-series survey of non-institutionalized older adults aged 65 or over residing in South Korea [21]. In abidance with the Elderly Welfare Act, the Korea Institute for Health and Social Affairs has been conducting this survey every three years since 2008 [22].

To inform welfare policies and respond to an aging society, this survey included questionnaire items regarding elderly individuals’ living arrangements, physical and mental health, healthcare use, and attitude toward death and LST [21]. No further ethical approval was required as informed consent was obtained from all participants and the data was publicly accessible [22].

The total survey population from the 2020 survey included 10,097 individuals. After excluding missing data (*N* = 181), responses from 9,916 participants (3,971 males; 5,945 females) comprised the study sample.

### Variables

The dependent variable was the attitude toward LST, which was asked through the question, ‘Would you prefer to receive life-sustaining treatment when you are unconscious or when staying alive is very difficult?’ It was a 5-point scale item, with 1 indicating ‘strongly agree’ and 5 indicating ‘strongly disagree’. Analyses were performed by categorizing 1 to 3 points as ‘agree’ and 4 to 5 points as ‘disagree’.

The main variable of interest in this study was the perceived life satisfaction of the elderly. Each participant was asked: ‘How satisfied are you with your current life in general?’ with the responses on a 5-point scale where 1 meant ‘very satisfied’ and 5 meant ‘very dissatisfied’. The responses were classified into two categories: 1 to 3 points meant ‘satisfied’ and 4 to 5 points indicated ‘dissatisfied’.

We controlled for covariates such as socioeconomic and health-related factors as potential confounders. Socioeconomic factors included sex, age, marital status, region, schooling years, and household income. Additionally, variables regarding health behavioural patterns included smoking, drinking, and physical exercise. The presence of the big five chronic diseases such as diabetes mellitus, cardiovascular disease, chronic respiratory disease, cancer, and stroke [23] and subjective health status was also corrected.

### Statistical analysis

Descriptive statistics were shown as frequencies (*N*) and percentages (%), and chi-squared test was conducted to investigate and compare the general characteristics of the study population. Subsequently, multivariable-adjusted Poisson regression models with robust variance were used to examine factors associated with attitude toward LST and calculate prevalence ratios (PR) and 95% confidence intervals (CI) [24–27]. For all analyses, we used SAS software, version 9.4 (SAS Institute Inc., Cary, NC, USA); *p*-values less than 0.05 were deemed statistically significant.

## Results

Table 1 shows the general characteristics of the population divided between those who were satisfied or dissatisfied with their current life. Of the 9,916 individuals included in this study, 3,971 (40.0%) were men and 5,945 (60.0%) were women. Among all participants, those who answered that they were satisfied with their current life accounted for 51.8% (*N* = 5,140), and those who answered that they were dissatisfied accounted for 48.2% (*N* = 4,776). In addition, 86.4% (*N* = 8,568) of the participants expressed opposition to LST, and only 13.6% (*N* = 1,348) were in favour of it.

**Table 1:** General characteristics of the study population

	Life satisfaction						
	Total		Satisfied^**a**^		Dissatisfied^**b**^		*P-value*
	N	%	N	%	N	%	
Characteristics	9916	100.0	5140	51.8	4776	48.2	
Sex							< .0001
Men	3971	40.0	2208	43.0	1763	36.9	
Women	5945	60.0	2932	57.0	3013	63.1	
Age							< .0001
65 ~ 69	3509	35.4	2200	42.8	1309	27.4	
70 ~ 74	2465	24.9	1283	25.0	1182	24.7	
75 ~ 79	1956	19.7	877	17.1	1079	22.6	
80 or over	1986	20.0	780	15.2	1206	25.3	
Marital status							< .0001
Married	5849	59.0	3308	64.4	2541	53.2	
Unmarried or Being separately	4067	41.0	1832	35.6	2235	46.8	
Region							< .0001
Urban	4308	43.4	2346	45.6	1962	41.1	
Rural	5608	56.6	2794	54.4	2814	58.9	
Schooling years							< .0001
0 ~ 6	4429	44.7	1844	35.9	2585	54.1	
7 ~ 12	4982	50.2	2917	56.8	2065	43.2	
13 or over	505	5.1	379	7.4	126	2.6	
Household income							< .0001
Tertile 1	3300	33.3	1482	28.8	1818	38.1	
Tertile 2	3307	33.4	1666	32.4	1641	34.4	
Tertile 3	3309	33.4	1992	38.8	1317	27.6	
Smoking							0.478
Yes	1088	11.0	575	11.2	513	10.7	
No	8828	89.0	4565	88.8	4263	89.3	
Drinking							< .0001
Seldom	6760	68.2	3291	64.0	3469	72.6	
Occasionally	2509	25.3	1515	29.5	994	20.8	
Frequently	647	6.5	334	6.5	313	6.6	
Physical exercise							< .0001
Yes	5186	52.3	2927	56.9	2259	47.3	
No	4730	47.7	2213	43.1	2517	52.7	
Big 5 chronic diseases^**c**^							< .0001
Yes	3169	32.0	1364	26.5	1805	37.8	
No	6747	68.0	3,776	73.5	2971	62.2	
Subjective health status							< .0001
Good	4939	49.8	3316	64.5	1623	34.0	
Bad	4977	50.2	1824	35.5	3153	66.0	

^a^ Those who answered 1 to 3 points on a 5-point scale question, “How satisfied are you with your current life in general?”

^b^ Those who answered 4 to 5 points to the same question as above

^c^ Diabetes mellitus, cardiovascular disease, chronic respiratory disease, cancer, and stroke

Table 2 presents the results of the multivariate Poisson regression models with robust variance, with attitudes in favour of LST as the outcome. As a result, the association between perceived life satisfaction and attitude toward LST among Korean older adults was identified. When all potential confounding variables were adjusted, the participants dissatisfied with their lives were more likely to agree to LST than the satisfied elderly, and the adjusted PR for men and women was found to be 1.52 (95% CI: 1.15–1.64) and 1.28 (95% CI: 1.09–1.51), respectively. Similarly, as shown in Supplementary table 1, in overall analysis of men and women, life satisfaction was found to be a factor related to the attitudes toward LST (Adjusted PR: 1.37; 95% CI: 1.22–1.55).

**Table 2:** Results of factors associated with attitudes in favor of life-sustaining treatment

Variables	Men						Women						
	Attitudes in favor of life-sustaining treatment						Attitudes in favor of life-sustaining treatment						
	N^**a**^	%^**b**^	Crude PR	95% CI	Adjusted PR	95% CI	N^**a**^	%^**b**^	Crude PR		95% CI	Adjusted PR	95% CI
Life satisfaction													
Satisfied	253	46.2	1.00		1.00		354	43.9	1.00			1.00	
Dissatisfied	295	53.8	1.38	(1.15-1.64	1.52	(1.26-1.83)	452	56.1	1.27		(1.09-1.47)	1.28	(1.09--1.51)
Age													
65 ~ 69	211	38.5	1.53	(1.14-2.05)	1.48	(1.06-2.05)	298	37.0	1.14	(0.93-1.40)		1.20	(0.93-1.55)
70 ~ 74	150	27.4	1.54	(1.14-2.08)	1.51	(1.09-2.08)	184	22.8	1.00	(0.80-1.26)		1.04	(0.81-1.32)
75 ~ 79	115	21.0	1.49	(1.07-2.07)	1.46	(1.05-2.02)	151	18.7	1.02	(0.79-1.31)		1.03	(0.80-1.33)
80 or over	72	13.1	1.00		1.00		173	21.5	1.00			1.00	
Marital status													
Married	444	81.0	1.00		1.00		359	44.5	1.00			1.00	
Unmarried or Being separately	104	19.0	0.83	(0.65-1.04)	0.84	(0.66-1.08)	447	55.5	0.93	(0.80-1.08)		0.88	(0.75-1.03)
Region													
Urban	276	50.4	1.00		1.00		411	51.0	1.00			1.00	
Rural	272	49.6	0.72	(0.61-0.86)	0.72	(0.60-0.85)	395	49.0	0.68	(0.59-0.79)		0.67	(0.58-0.78)
Schooling years													
0 ~ 6	167	30.5	1.78	(1.17-2.71)	1.85	(1.19-2.89)	461	57.2	1.48	(0.82-2.65)		1.50	(0.82-2.73)
7 ~ 12	354	64.6	1.92	(1.29-2.86)	1.72	(1.15-2.57)	333	41.3	1.48	(0.83-2.67)		1.41	(0.78-2.56)
13 or over	27	4.9	1.00		1.00		12	1.5	1.00			1.00	
Household income													
Tertile 1	118	21.5	0.80	(0.63-1.02)	0.85	(0.65-1.09)	356	44.2	1.03	(0.86-1.22)		1.08	(0.89-1.30)
Tertile 2	213	38.9	0.95	(0.78-1.16)	0.98	(0.80-1.20)	221	27.4	0.88	(0.73-1.07)		0.91	(0.75-1.11)
Tertile 3	217	39.6	1.00		1.00		229	28.4	1.00			1.00	
Smoking													
Yes	137	25.0	1.10	(0.90-1.34)	0.93	(0.76-1.15)	14	1.7	0.77	(0.44-1.35)		0.76	(0.45-1.31)
No	411	75.0	1.00		1.00		792	98.3	1.00			1.00	
Drinking													
Seldom	213	38.9	1.00		1.00		628	77.9	1.00			1.00	
Occasionally	266	48.5	1.59	(1.32-1.93)	1.52	(1.25-1.85)	167	20.7	1.44	(1.21-1.72)		1.45	(1.20-1.74)
Frequently	69	12.6	1.23	(0.92-1.64)	1.10	(0.82-1.48)	11	1.4	0.74	(0.41-1.37)		0.84	(0.46-1.53)
Physical exercise													
Yes	292	53.3	1.00		1.00		372	46.2	1.00			1.00	
No	256	46.7	1.18	(0.99-1.40)	1.18	(0.99-1.40)	434	53.9	1.27	(1.09-1.47)		1.24	(1.07-1.45)
Big 5 chronic diseases													
Yes	162	29.6	0.88	(0.72-1.06)	0.91	(0.74-1.11)	251	31.1	1.02	(0.87-1.20)		1.00	(0.84-1.18)
No	386	70.4	1.00		1.00		555	68.9	1.00			1.00	
Subjective health status													
Good	313	57.1	1.00		1.00		345	42.8	1.00			1.00	
Bad	235	42.9	0.91	(0.76-1.09)	0.91	(0.74-1.11)	461	57.2	1.11	(0.96-1.29)		1.11	(0.93-1.31)

^a^The number of respondents who answered 1 to 3 points on a 5-point scale question, ‘What do you think about life-sustaining treatment even though you are unconscious or difficult to survive?’

^b^In the column, the percentage of the answer 1 to 3 points to the question of attitudes toward life-sustaining treatment

^*^: *p*-value < 0.05

Additionally, we conducted subgroup analysis stratified by socioeconomic factors, such as age, region, schooling years, and household income, because it was expected that factors would affect the perception of LST among the elderly. As noted in Table 3, significant associations were prominent in the relatively young elderly aged 65 to 74 (Men, Adjusted PR: 1.71, 95% CI: 1.37–2.12; Women, Adjusted PR: 1.44, 95% CI: 1.19–1.74), and the urban dweller group (Men, Adjusted PR: 2.20, 95% CI: 1.71–2.82; Women, Adjusted PR: 1.34, 95% CI: 1.06–1.70). In the case of the elderly with a long schooling period of more than 7 years, it was confirmed that the probability of favouring LST was statistically significantly higher when they were dissatisfied with their life (Men, Adjusted PR: 1.78, 95% CI: 1.44–2.20; Women, Adjusted PR: 1.42, 95% CI: 1.13–1.80). Similarly, the elderly with the highest income level were found to be more likely to agree to LST when they felt dissatisfied with their life. The statistical significance of the tertile 3 group (highest earner) was found to be common in all sexes (Men, Adjusted PR: 1.99, 95% CI: 1.49–2.66; Women, Adjusted PR: 1.72, 95% CI: 1.30–2.27).

**Table 3:** Results of subgroup analysis stratified by socioeconomic factors

Variables	Men				Women				
	Attitudes in favor of life-sustaining treatment				Attitudes in favor of life-sustaining treatment				
	Life Satisfaction				Life Satisfaction				
	Satisfied	Dissatisfied			Satisfied		Dissatisfied		
	APR^a^	APR^a^	95% CI		APR^a^		APR^a^		95% CI
Age									
65 ~ 74	1.00	1.71	(1.37-2.12)		1.00		1.44		(1.19-1.74)
85 or over	1.00	1.30	(0.93-1.81)		1.00		1.06		(0.79-1.41)
Region									
Urban	1.00	2.20	(1.71-2.82)	1.00		1.34		(1.06-1.70)	
Rural	1.00	1.08	(0.83-1.42)	1.00		1.23		(0.99-1.53)	
Schooling years									
0 ~ 6	1.00	1.08	(0.76-1.54)	1.00		1.15		(0.92-1.43)	
7 or over	1.00	1.78	(1.44-2.20)	1.00		1.42		(1.13-1.80)	
Household income									
Tertile 1	1.00	1.32	(0.85-2.05)	1.00		1.09		(0.88-1.38)	
Tertile 2	1.00	1.24	(0.93-1.64)	1.00		1.09		(0.80-1.49)	
Tertile 3	1.00	1.99	(1.49-2.66)	1.00		1.72		(1.30-2.27)	

^a^ APRs (Adjusted prevalence ratios) were adjusted for other covariates, respectively

## Discussion

The Well-dying Act that allows patients with no possibility of rehabilitation to withhold or withdraw LST with their own decision or family consent has been enforced in Korea since 2018 [5, 6]. Although still in the transitional phase, 86.4% of the participants expressed opposition to LST, and with only 13.6% in favour of it. After adjusting several covariates such as socioeconomic and health-related factors, it was found that elderly people’s satisfaction with life was related to their attitude toward LST.

For patients on the verge of death, LST is a self-determinant right, so it is difficult to say which decision is more correct, and it must be interpreted carefully. In this context, this study focused on examining factors affecting elderly individuals’ attitude toward LST at the time of end-of-life. Summarizing the key findings of our study, the elderly who feel satisfied with life are more likely to withhold or withdraw LST by themselves according to the purpose of the Life-Sustaining Treatment Decisions Act. Perhaps we would expect that people who are dissatisfied with their current life will stop LST, but the opposite association was drawn. These results suggest that the elderly who are satisfied with their life are more likely to make a decision to withhold or withdraw the LST by writing an advance decision on LST based on more rational thinking, not that they are no longer willing to maintain their lives. On the other hand, the elderly who are dissatisfied with their current life may have more regrets for the rest of their life.

One interesting finding was that the presence or absence of big 5 chronic diseases was not a statistically significant factor influencing LST preference. This suggests that, in addition to health status, various socioeconomic factors of the elderly have a greater influence on LST determination. In addition, if the elderly of relatively young age group, living in an urban area, a long schooling years, or a high household income were dissatisfied with their life, they were more likely to approve of LST. These findings suggest that life satisfaction is an important factor in exercising the right to determine LST at end-of-life, even if socioeconomic conditions are relatively better. Therefore, interventions to increase elderly's life satisfaction will be needed so that elderly patients on the verge of death can make their own decisions about LST.

There have been several previous studies and interventions on the attitude toward end-of-life care [20] and LST [13, 17, 28, 29] in general patients and the elderly. Similar to this study, some studies investigated the effects of depression [30] and perceived quality of life [31] on the decision regarding LST in the elderly. They have reported that the elderly who were not depressed or had higher perceived quality of life were more likely to withdraw from LST, which is consistent with our main findings. However, in South Korea, as the Well-Dying Act and Life-Sustaining Treatment Decision Act were implemented fairly recently, most of the preceding studies discussed the implication [32] and current status of the Act [1, 5, 6], so there was an insufficient number of prior studies to which we could refer. Therefore, our study is different in that it explored factors affecting LST preference, which is emerging as an important issue in the rapidly aging population, using the most recent survey data for the elderly officially conducted in Korea.

This study had certain limitations. First, issues related to LST may be more focused on patients with severe diseases or the elderly who are on the verge of death, but it was not possible to separate these subjects and conduct additional analysis. To compensate for this limitation, the prevalence of the big five chronic diseases defined by the World Health Organization [23] was corrected as a covariate. Second, since this study was a cross-sectional study based on the latest 2020 data, the association was confirmed, but causality was not confirmed. Therefore, an additional longitudinal study on changes in participants’ attitudes towards LST should be conducted in severely ill patients or the elderly. Third, even after adjusting for numerous covariates that may affect the dependent variable, there will still be potential confounding effects from the unmeasurable variables.

## Conclusions

Our findings identified a positive association between elderly people’s satisfaction with their current lives and their attitude toward withdrawing LST. Since it is desirable that the forgoing or withdrawal of LST depend on the decisions of the elderly and patients themselves, ways to improve perceived life satisfaction should be sought so that the right to self-determination can be exercised correctly.

## Supplementary Information

**Additional file 1:** **Supplementary Table1.** Results of factors associated with attitudes in favor of life-sustaining treatment (overall analysis of men and women).

## Data Availability

The data is publicly accessible on the website of Korea Institute for Health and Social Affairs (https://www.kihasa.re.kr/).
