# Supplementary material for: Association of perceived life satisfaction with attitudes toward life-sustaining treatment among the elderly in South Korea: a cross-sectional study
Source: BMC Palliat Care. 2022 Oct 17;21:184. doi: 10.1186/s12904-022-01072-6 (PMC9575263; doi:10.1186/s12904-022-01072-6)
Supplement: Supplementary file 1 — Additional file 1: Supplementary Table1. Results of factors associated with attitudes in favor of life-sustaining treatment (overall analysis of men and women). [file 12904_2022_1072_MOESM1_ESM.docx]

| **Supplementary table1. Results of factors associated with attitudes in favor of life-sustaining treatment (overall analysis of men and women)** | | | | | | | | | | | | |
| --- | --- | --- | --- | --- | --- | --- | --- | --- | --- | --- | --- | --- |
|  | | **Attitudes in favor of life-sustaining treatment** | | | | | | | | | | |
|  |  | **N^a^** | **%^b^** | **Crude PR** | **95% CI** | | |  | **Adjusted PR** | **95% CI** | | |
| **Life satisfaction** | |  |  |  |  |  |  |  |  |  |  |  |
|  | Satisfied | 5140 | 51.8 | 1.00 |  |  |  |  | 1.00 |  |  |  |
|  | Dissatisfied | 4476 | 48.2 | 1.31 | (1.17 | - | 1.47) |  | 1.37 | (1.22 | - | 1.55) |
| **Sex** | |  |  |  |  |  |  |  |  |  |  |  |
|  | Men | 3971 | 40.0 | 1.00 |  |  |  |  | 1.00 |  |  |  |
|  | Women | 5945 | 60.0 | 1.00 | (0.89 | - | 1.12) |  | 1.06 | (0.93 | - | 1.22) |
| **Age** | |  |  |  |  |  |  |  |  |  |  |  |
|  | 65~69 | 3509 | 35.4 | 1.27 | (1.07 | - | 1.50) |  | 1.27 | (1.04 | - | 1.54) |
|  | 70~74 | 2465 | 24.9 | 1.19 | (0.99 | - | 1.42) |  | 1.20 | (0.99 | - | 1.45) |
|  | 75~79 | 1956 | 19.7 | 1.18 | (0.97 | - | 1.43) |  | 1.18 | (0.97 | - | 1.43) |
|  | 80 or over | 1986 | 20.0 | 1.00 |  |  |  |  | 1.00 |  |  |  |
| **Marital status** | |  |  |  |  |  |  |  |  |  |  |  |
|  | Married | 5849 | 59.0 | 1.00 |  |  |  |  | 1.00 |  |  |  |
|  | Unmarried or Being separately | 4067 | 41.0 | 0.81 | (0.65 | - | 1.03) |  | 0.89 | (0.78 | - | 1.01) |
| **Region** | |  |  |  |  |  |  |  |  |  |  |  |
|  | Urban | 4308 | 43.4 | 1.00 |  |  |  |  | 1.00 |  |  |  |
|  | Rural | 5608 | 56.6 | 0.70 | (0.62 | - | 0.78) |  | 0.69 | (0.62 | - | 0.78) |
| **Schooling years** | |  |  |  |  |  |  |  |  |  |  |  |
|  | 0~6 | 4429 | 44.7 | 1.70 | (1.22 | - | 2.38) |  | 1.75 | (1.24 | - | 2.48) |
|  | 7~12 | 4982 | 50.2 | 1.77 | (1.27 | - | 2.46) |  | 1.65 | (1.18 | - | 2.30) |
|  | 13 or over | 505 | 5.1 | 1.00 |  |  |  |  | 1.00 |  |  |  |
| **Household income** | |  |  |  |  |  |  |  |  |  |  |  |
|  | Tertile 1 | 3300 | 33.3 | 0.94 | (0.82 | - | 1.08) |  | 0.98 | (0.85 | - | 1.14) |
|  | Tertile 2 | 3307 | 33.4 | 0.92 | (0.80 | - | 1.05) |  | 0.94 | (0.81 | - | 1.08) |
|  | Tertile 3 | 3309 | 33.4 | 1.00 |  |  |  |  | 1.00 |  |  |  |
| **Smoking** | |  |  |  |  |  |  |  |  |  |  |  |
|  | Yes | 1088 | 11.0 | 1.05 | (0.88 | - | 1.25) |  | 0.93 | (0.77 | - | 1.13) |
|  | No | 8828 | 89.0 | 1.00 |  |  |  |  | 1.00 |  |  |  |
| **Drinking** | |  |  |  |  |  |  |  |  |  |  |  |
|  | Seldom | 6760 | 68.2 | 1.00 |  |  |  |  | 1.00 |  |  |  |
|  | Occasionally | 2509 | 25.3 | 1.44 | (1.28 | - | 1.63) |  | 1.49 | (1.30 | - | 1.69) |
|  | Frequently | 647 | 6.5 | 1.03 | (0.81 | - | 1.32) |  | 1.06 | (0.82 | - | 1.36) |
| **Physical exercise** | |  |  |  |  |  |  |  |  |  |  |  |
|  | Yes | 5186 | 52.3 | 1.00 |  |  |  |  | 1.00 |  |  |  |
|  | No | 4730 | 47.7 | 1.23 | (1.09 | - | 1.37) |  | 1.22 | (1.08 | - | 1.36) |
| **Big 5 chronic diseases** | |  |  |  |  |  |  |  |  |  |  |  |
|  | Yes | 3169 | 32.0 | 0.96 | (0.84 | - | 1.08) |  | 0.96 | (0.84 | - | 1.09) |
|  | No | 6747 | 68.0 | 1.00 |  |  |  |  | 1.00 |  |  |  |
| **Subjective health status** | |  |  |  |  |  |  |  |  |  |  |  |
|  | Good | 4939 | 49.8 | 1.00 |  |  |  |  | 1.00 |  |  |  |
|  | Bad | 4977 | 50.2 | 1.02 | (0.91 | - | 1.14) |  | 1.02 | (0.89 | - | 1.16) |
| ^a^ The number of respondents who answered 1 to 3 points on a 5-point scale question, “What do you think about life-sustaining treatment even though you are unconscious or difficult to survive?” | | | | | | | | | | | | |
| ^b^ In the column, the percentage of the answer 1 to 3 points to the question of attitudes toward life-sustaining treatment | | | | | | | | | | |  |  |
| * : p-value<0.05 | |  |  |  |  |  |  |  |  |  |  |  |
